# Supplementary material for: Retinoic acid signaling modulation guides in vitro specification of human heart field-specific progenitor pools
Source: Nat Commun. 2023 Apr 3;14:1722. doi: 10.1038/s41467-023-36764-x (PMC10070453; doi:10.1038/s41467-023-36764-x)
Supplement: Supplementary file 1 — Supplementary Information [file 41467_2023_36764_MOESM1_ESM.pdf]

Supplementary Information for

Retinoic acid signaling modulation guides *in vitro* specification of human heart field-specific progenitor pools

Dorota Zawada[1,2,3‡], Jessica Kornherr[1,2,3‡], Anna B. Meier[1,2,3‡], Gianluca Santamaria[1,2,3,4‡], Tatjana Dorn[1,2,3], Monika Nowak-Imialek[1,2,3], Daniel Ortmann[5,6], Fangfang Zhang[1,2,3], Mark Lachmann[1,2], Martina Dreßen[7], Mariaestela Ortiz[5], Victoria L. Mascetti[8], Stephen C. Harmer[9], Muriel Nobles[10], Andrew Tinker[10], Maria Teresa De Angelis[1,2,4], Roger A. Pedersen[11], Phillip Grote[12,13], Karl-Ludwig Laugwitz[1,2*], Alessandra Moretti[1,2,3,14*], Alexander Goedel[1,15*]

Affiliations

[1]First Department of Medicine, Cardiology, Klinikum rechts der Isar, Technical University of Munich, School of Medicine and Health, Munich, Germany.

[2]German Center for Cardiovascular Research (DZHK), Munich Heart Alliance, Munich, Germany.

[3]Regenerative Medicine in Cardiovascular Diseases, First Department of Medicine, Klinikum rechts der Isar, Technical University of Munich, School of Medicine and Health, Munich, Germany.

[4]Department of Experimental and Clinical Medicine, University “Magna Graecia”, Catanzaro, Italy.

[5]Department of Surgery, University of Cambridge, Cambridge, UK.

[6]Wellcome-MRC Cambridge Stem Cell Institute, Jeffrey Cheah Biomedical Centre, University of Cambridge, Cambridge, UK.

[7]German Heart Center Munich, Department of Cardiovascular Surgery, Institute Insure - Technical University of Munich, School of Medicine and Health, Munich, Germany.

[8]Bristol Heart Institute, Bristol Medical School, Translational Health Sciences, Bristol, UK.

[9]School of Physiology, Pharmacology and Neuroscience, University of Bristol, Biomedical Sciences Building, University Walk, Bristol, UK

[10]Clinical Pharmacology & Precision Medicine, William Harvey Research Institute, Barts and the London School of Medicine and Dentistry, Queen Mary University of London, London, UK.

[11]Department of Obstetrics and Gynecology, Stanford School of Medicine, Stanford University, USA.

[12]Georg-Speyer-Haus, Institute for Tumor Biology and Experimental Therapy, Frankfurt am Main, Germany.

[13]Institute of Cardiovascular Regeneration, Centre for Molecular Medicine, Goethe University, Frankfurt am Main, Germany.

[14]Department of Surgery, Yale University School of Medicine; New Haven, USA

[15]Department of Cell and Molecular Biology, Karolinska Institute, Stockholm, Sweden.

[‡] These authors contributed equally.

*Corresponding authors: laugwitz@mytum.de, amoretti@mytum.de, alexander.goedel@tum.de

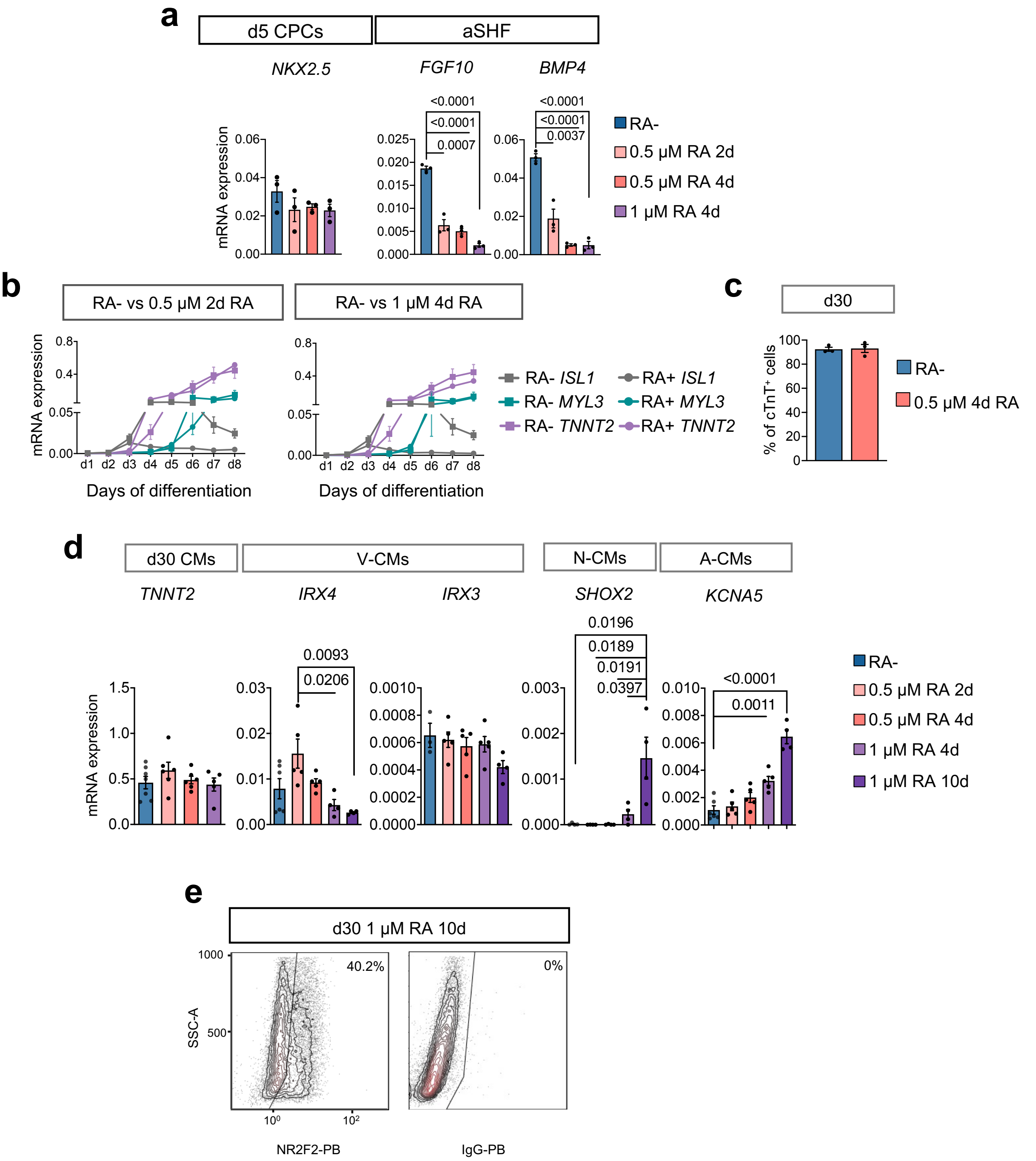

Supplementary Figure 1: Manipulation of RA signalling allows the selective enrichment for distinct pools of human heart field-like progenitors and their derivatives. (related to Figure 1). (a) mRNA expression of cardiovascular progenitors (CPCs) marker *NKX2.5* and anterior second heart field (aSHF) markers *FGF10* and *BMP4* at day 5 in the indicated differentiation conditions. Data are mean ± SEM; n = 3 independent experiments. Exact p-values of unpaired two-tailed *t*-test are shown. **(b)** Time course of mRNA expression of *ISL1*, *MYL3*, *TNNT2* relative to *GAPDH* during differentiation without retinoic acid (RA-) compared to differentiation with different dosage and time of RA (RA+). Data are mean n = 2 independent samples/time point except for: RA- data are mean ± SEM n = 3. **(c)** Quantification of flow

cytometry analysis of cells stained positive for cTnT at day 30 of differentiation without retinoic acid (RA-) and with 0.5 μM RA for 4d. Data are mean ± SEM; n = 3 independent experiments. **(d)** mRNA expression of *TNNT2* and markers of ventricular cardiomyocytes (V-CMs; *IRX4*, *IRX3*), atrial cardiomyocytes (A-CMs; *KCNA5*) and nodal cardiomyocytes (N-CMs; *SHOX2*) relative to *GAPDH* (for *TNNT2*) or *GAPDH* and *TNNT2* (for other genes) at day 30 of the indicated differentiation conditions. Data are mean ± SEM; n = 4 independent experiments unless RA- *TNNT2* n = 7, *IRX4* and *KCNA5* n = 6, *IRX3* n = 3; RA+ (0.5 μM 2d and 4d) *IRX3*, *IRX4*, *KCNA5* n = 5, *TNNT2* n = 6; RA+ (1 μM 4d) *TNNT2* n = 6, *IRX3* and *KCNA5* n = 5. Exact p-values of unpaired two-tailed *t*-test are shown**. (e)** Representative plots of flow cytometry analysis of cells stained positive for NR2F2 or IgG (negative control) at day 30 of 1 μM 10d RA differentiation. Related to Figure 2c. Source data are provided as a Source Data file.

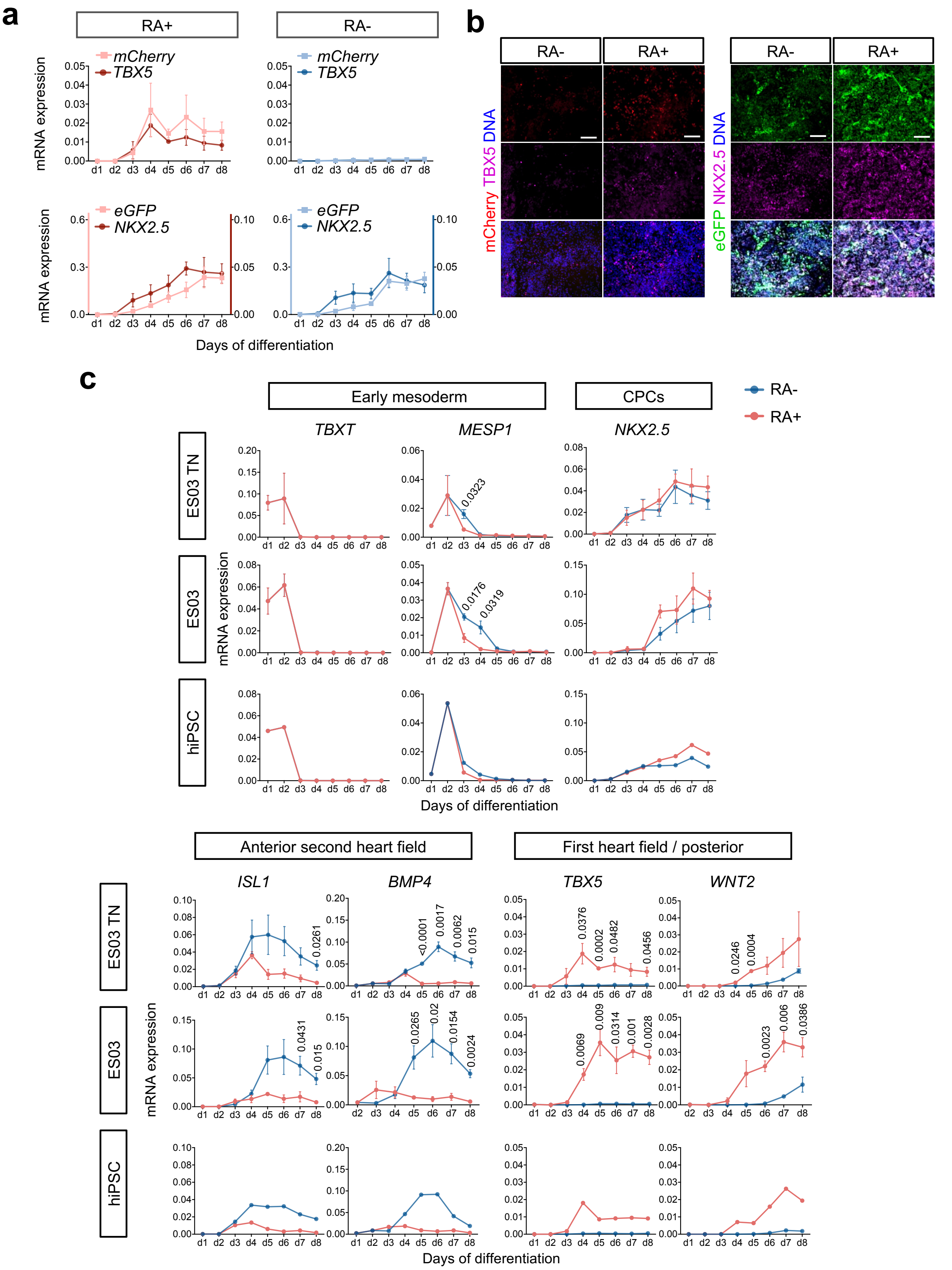

Supplementary Figure 2: Early steps of cardiac differentiation are reproducible across human pluripotent stem cells lines (related to Figure 3). (a) Time course mRNA expression of *mCherry* and *TBX5* as well as *eGFP* and *NKX2.5* (relative to *GAPDH)* during differentiation without retinoic acid (RA-) and with 0.5 µM RA for 4d (RA+). Data are mean ± SEM; n = 3

independent samples/time point. **(b)** Representative immunofluorescence images of cells at day 10 of differentiation without retinoic acid (RA-) and with 0.5 μM RA for 4d (RA+) stained for **(left)** mCherry (red) and TBX5 (magenta) and **(right)** eGFP (green) and NKX2.5 (magenta). Nuclei were counterstained with Hoechst-33258 (blue). Scale bars: 100 μm. **(c)** mRNA expression of *TBXT, MESP1, NKX2.5, ISL1, BMP4*, *TBX5* and *WNT2* relative to *GAPDH* during differentiation without retinoic acid (RA-) and with 0.5 μM RA for 4d (RA+) for 3 cell lines: ES03 TBX5-mCherry NKX2.5-eGFP (ES03 TN); parental line ES03 and independent hiPSC line. For ES03 TN and ES03 data are mean ± SEM and n = 3 independent samples/time point for; for hiPSC data are mean n = 2. Exact p-values of unpaired two-tailed *t*-test are shown**.** CPCs: cardiovascular progenitors.

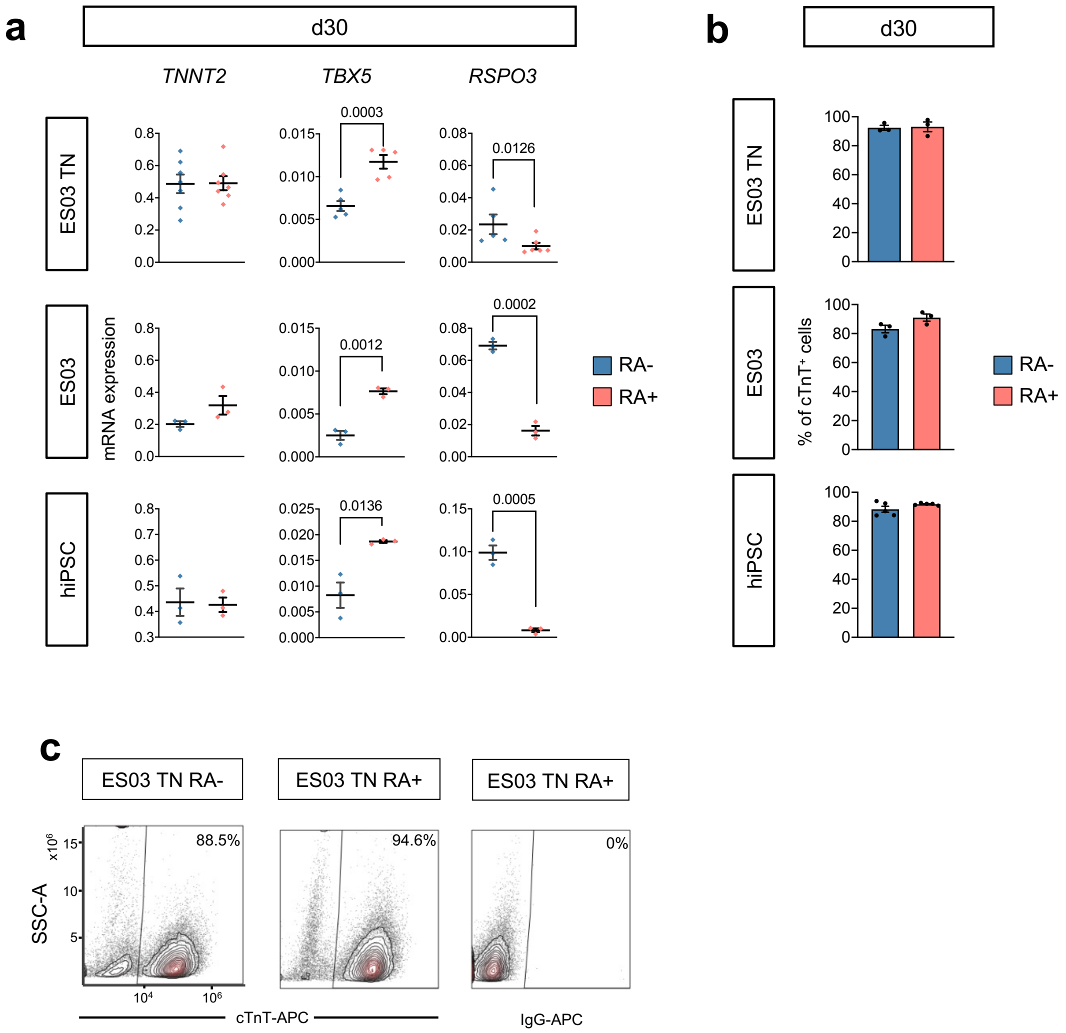

Supplementary Figure 3: Results of cardiac differentiation at day 30 are reproducible across human pluripotent stem cells lines (related to Figure 3). (a) mRNA expression of *TNNT2*, *TBX5*, and *RSPO3* at day 30 of differentiation without retinoic acid (RA-) and with 0.5 µM RA for 4d (RA+) for the 3 hPSC lines. Data are mean ± SEM; for ES03 TN n = 6 independent experiments except for: *TNNT2* RA- n = 7, *RSPO3* RA+ n = 5, *TBX5* RA- n = 5; for ES03 and hiPSC n = 3. mRNA expression relative to *GAPDH* for *TNNT2* or relative to *GAPDH* and *TNNT2* for other genes. Exact p-values of unpaired two-tailed *t*-test are shown**. (b)** Quantification of flow cytometry analysis of cells stained positive for cTnT at day 30 of differentiation without retinoic acid (RA-) and with 0.5 µM RA for 4d (RA+) for the 3 hPSC lines. Data are mean ± SEM; n = 3 independent experiments for ES03 and ES03 TN, and n = 5 for hiPSC. **(c)** Representative plots of flow cytometry analysis of cells stained positive for cTnT or IgG (negative control) at day 30 of differentiation without retinoic acid (RA-) and with 0.5 µM RA for 4d (RA+) of ES03 TN cell line. Related to Supplementary Figure 3b and 10a. Source data are provided as a Source Data file.

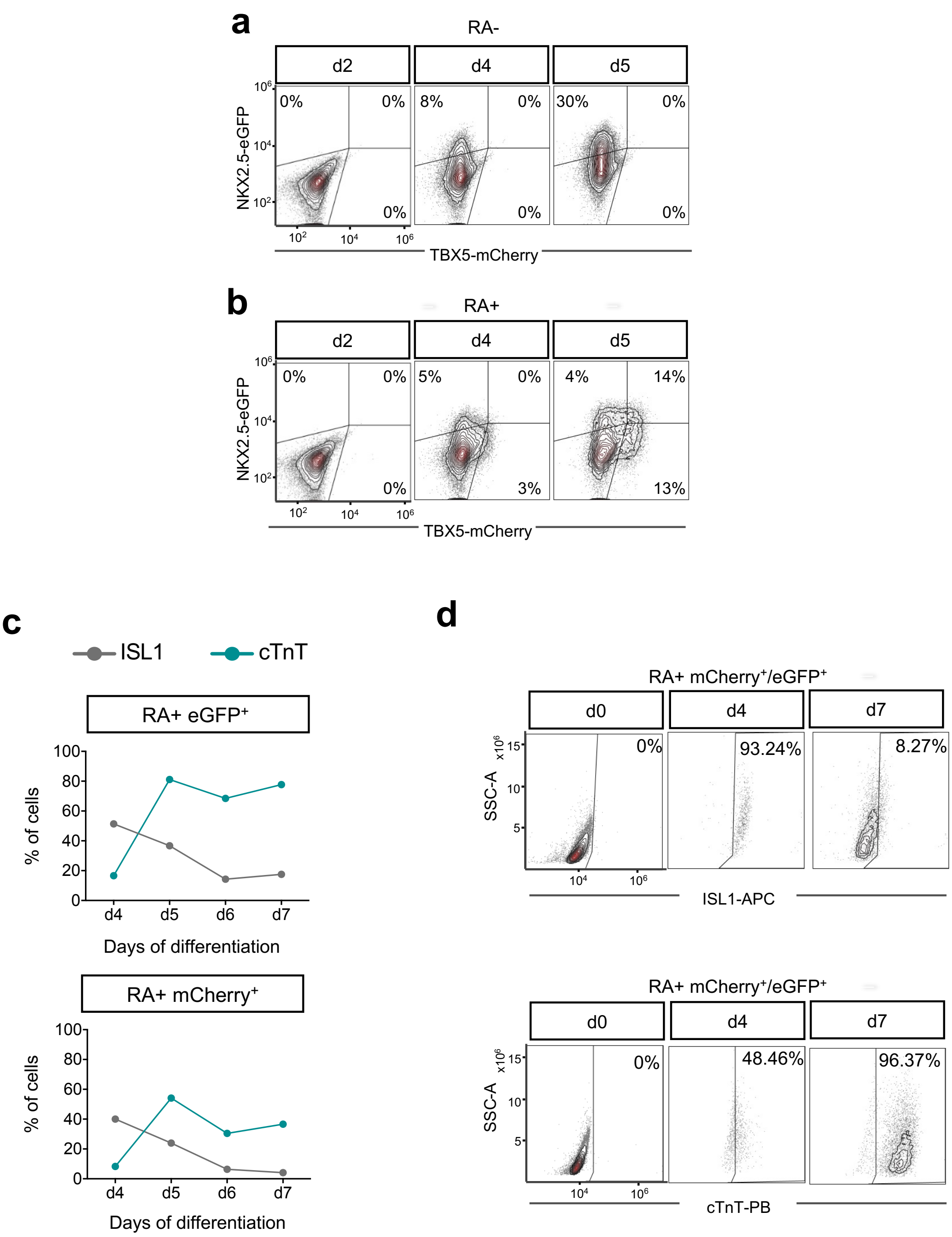

Supplementary Figure 4: Generation of TBX5^{mCherry} and NKX2.5^{eGFP} human embryonic stem cell line allows to track the appearance of distinct cardiovascular progenitor pools (related to Figure 3). (a, b) Representative plots of live flow cytometry time course analysis of cells expressing mCherry (TBX5) and eGFP (NKX2.5) during **(a)** RA- and **(b)** 0.5 µM 4d RA differentiation. Related to Figure 3c. **(c)** Quantification of flow cytometry time course analysis of cells stained positive for ISL1 and cTnT within mCherry$^+$ (TBX5$^+$) and eGFP$^+$ (NKX2.5$^+$) populations from 0.5 µM 4d RA differentiation. Data are mean; n = 2 independent experiments. **(d)** Representative plots of flow cytometry analysis of cells stained for cTnT, ISL1 and IgG

(negative control) during RA- and 0.5 μM 4d RA differentiation. Related to Figure 3d and Supplementary Figure 4c. Source data are provided as a Source Data file.

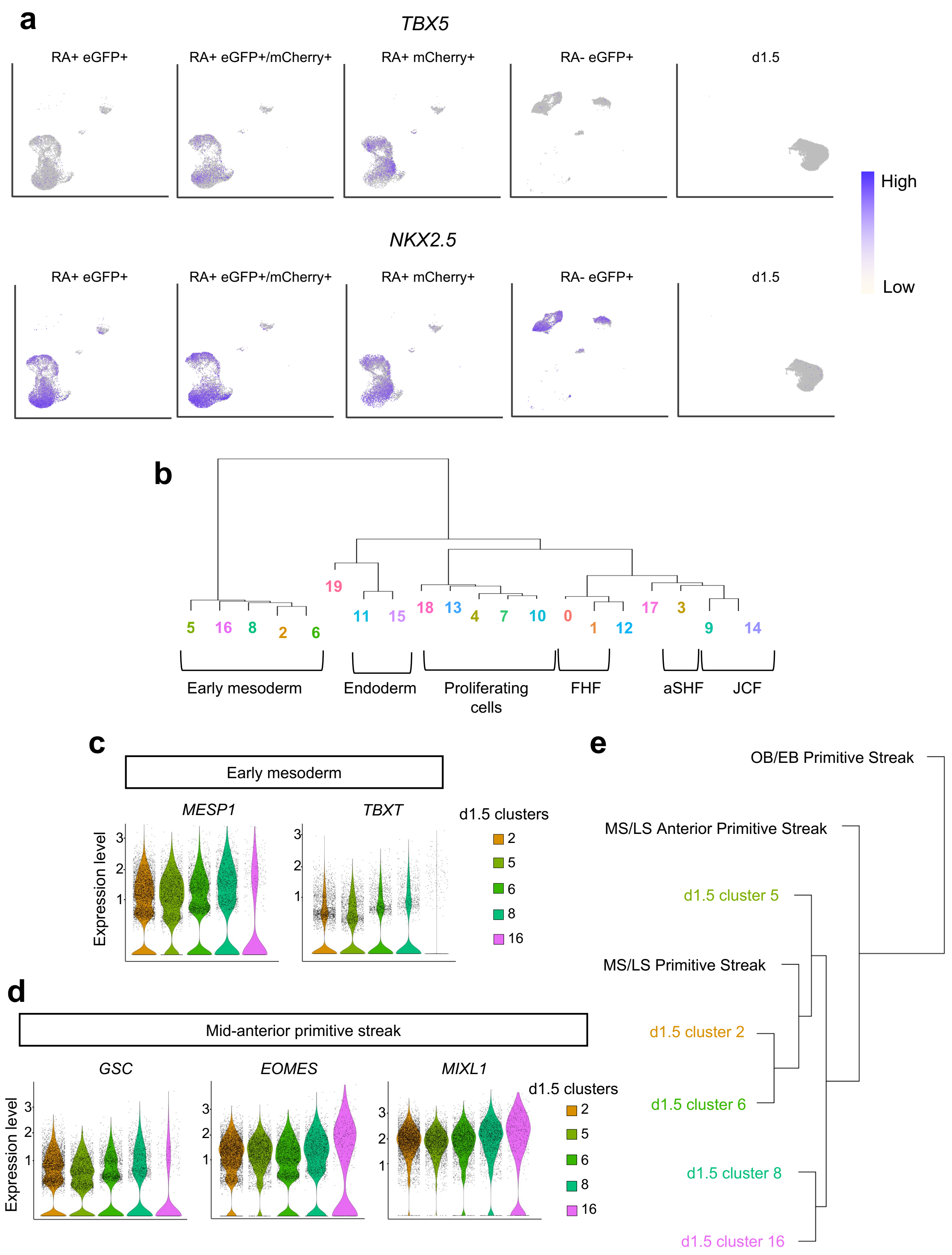

Supplementary Figure 5: Cells at day 1.5 correspond to mesodermal progenitors emerging from the mid-anterior primitive streak (related to Figure 3). (a) Feature plots showing expression of *TBX5* and *NKX2.5* in the indicated cells sorted at day 4.5 or collected at day 1.5. UMAP plot shown in Fig. 3f. **(b)** Dendrogram showing hierarchical clustering of the

averaged, corrected, normalized expression values per indicated day 1.5 and day 4.5 scRNA-seq clusters. UMAP plot shown in Fig. 3f. **(c)** Violin plots showing the expression levels of early mesoderm genes (*MESP1*, *TBXT*) at day 1.5 in the scRNA-seq clusters shown in Fig. 3f. **(d)** Violin plots showing expression levels of genes defining the mid-anterior primitive streak stage (*GSC*, *EOMES*, *MIXL1*) at day 1.5 in the scRNA-seq clusters shown in Fig. 3f. **(e)** Dendrogram showing hierarchical clustering of the averaged, corrected, normalized expression values per clusters after integration of indicated scRNA-seq clusters with scRNA-seq dataset of mouse gastrulation.[4] MS-LS: middle streak - late streak; OB-EB: no-bud - early bud.

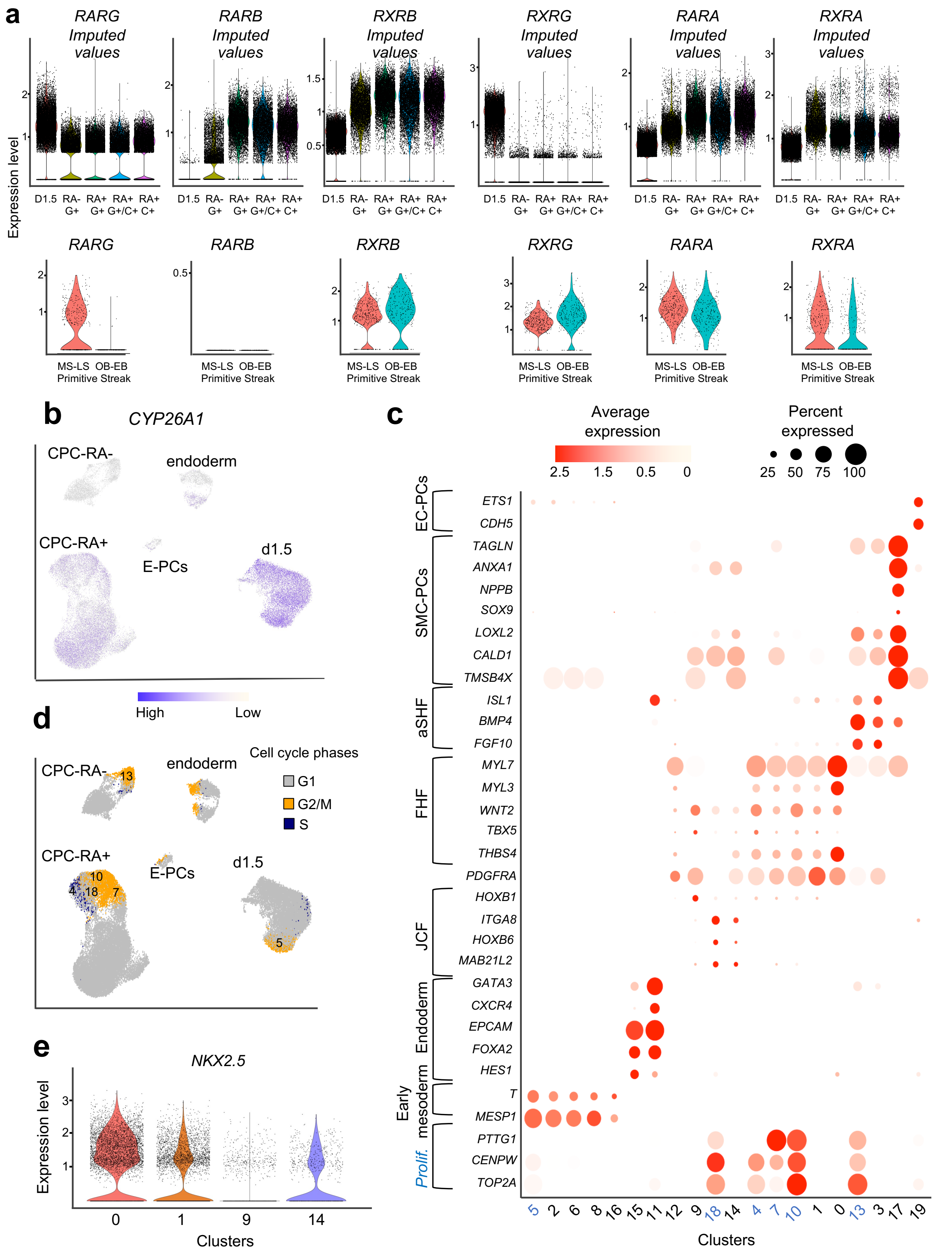

Supplementary Figure 6: Characterization of human cardiovascular progenitors (related to Figure 3). (a) Violin plots showing expression levels of genes encoding for retinoic acid receptors in **(top)** populations of cells collected at day 1.5 and sorted at day 4.5 for mCherry (TBX5) and eGFP (NKX2.5) expression and **(bottom)** clusters from scRNA-seq dataset of

mouse gastrulation[4]. UMAP plot shown in 3f. G+: eGFP$^+$; C+: mCherry$^+$. MS-LS: middle streak - late streak; OB-EB: no-bud - early bud. Missing values were imputed using ALRA (see Methods). **(b)** Feature plot showing the expression of *CYP26A1* at day 1.5 and day 4.5 in the UMAP plot shown in Fig. 3f. **(c)** Dot plot showing the expression level of selected differentially expressed genes in the indicated clusters at day 1.5 and day 4.5 in the UMAP plot shown in Fig. 3f. Proliferating clusters are in blue. **(d)** Feature plot showing the expression of gene signatures specific of cell cycles phases – G2/M phase (yellow), S phase (blue), G1 (grey) – at day 1.5 and day 4.5 in the UMAP plot shown in Fig. 3f; main cell types are annotated. Clusters of proliferating cells are indicated. **(e)** Violin plot showing expression of *NKX2.5* in the indicated clusters at day 1.5 and day 4.5 in the UMAP plot shown in Fig. 3f.

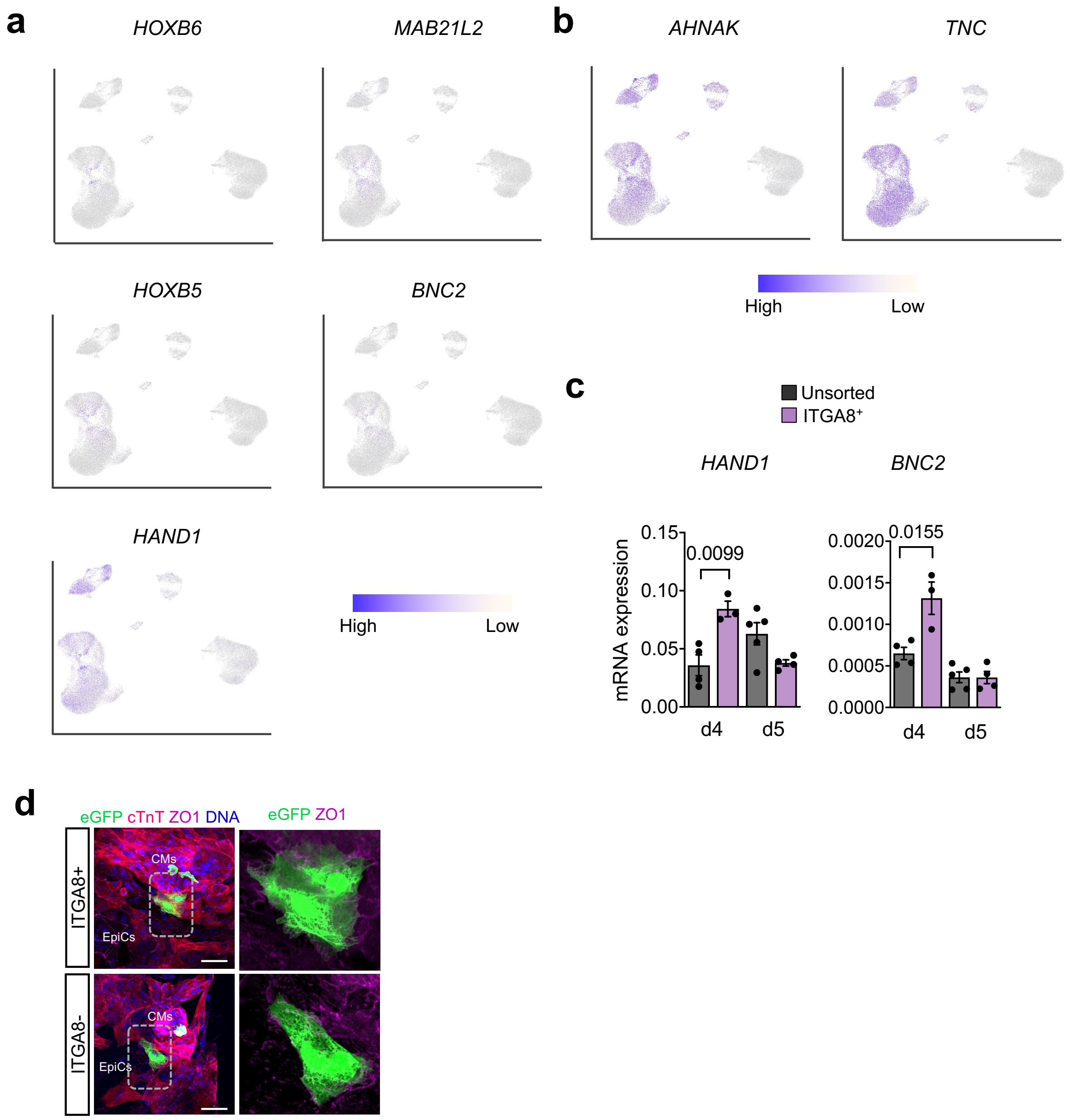

Supplementary figure 7: Characterization of human JCF-like progenitors (related to Figure 4). (a) Feature plots showing expression levels of key JCF markers (*HOXB6, MAB21L2, HOXB5, BNC2, HAND1*) at day 1.5 and day 4.5 in the UMAP plot shown in Fig. 3f. **(b)** Feature plots showing the expression levels of two cell surface marker candidates (*AHNAK, TNC*) of JCF identified through comparative differential gene expression analysis between the mouse dataset of Tyser et al.[6] and our dataset. **(c)** mRNA expression of the JCF markers *HAND1* and *BNC2* relative to *GAPDH* in day 4 and day 5 cells flow cytometry-based sorted for ITGA8 (APC) and unsorted cells during 0.5 µM 4d RA+ differentiation. Data are mean ± SEM; n = 4 independent experiments except for: sorted day 4 n = 3, unsorted day 5 n = 5. Exact p-values of unpaired two-tailed *t*-test are shown. **(d)** **(left)** Representative images of cells stained for eGFP (green) as well as cTnT (red) and ZO1 (magenta) 10 days after replating of

eGFP^{+}/ITGA8^{+} or eGFP^{+}/ITGA8^{-} sorted progenitors at day 4.5 of differentiation with 0.5 μM RA for 4d examined over 3 independent experiments. Scale bar = 50 μm. Nuclei were counterstained with Hoechst-33258 (blue). **(right)** A high magnification of boxed regions from the corresponding left panels showing cells stained for eGFP (green) and ZO1 (magenta). Source data are provided as a Source Data file.

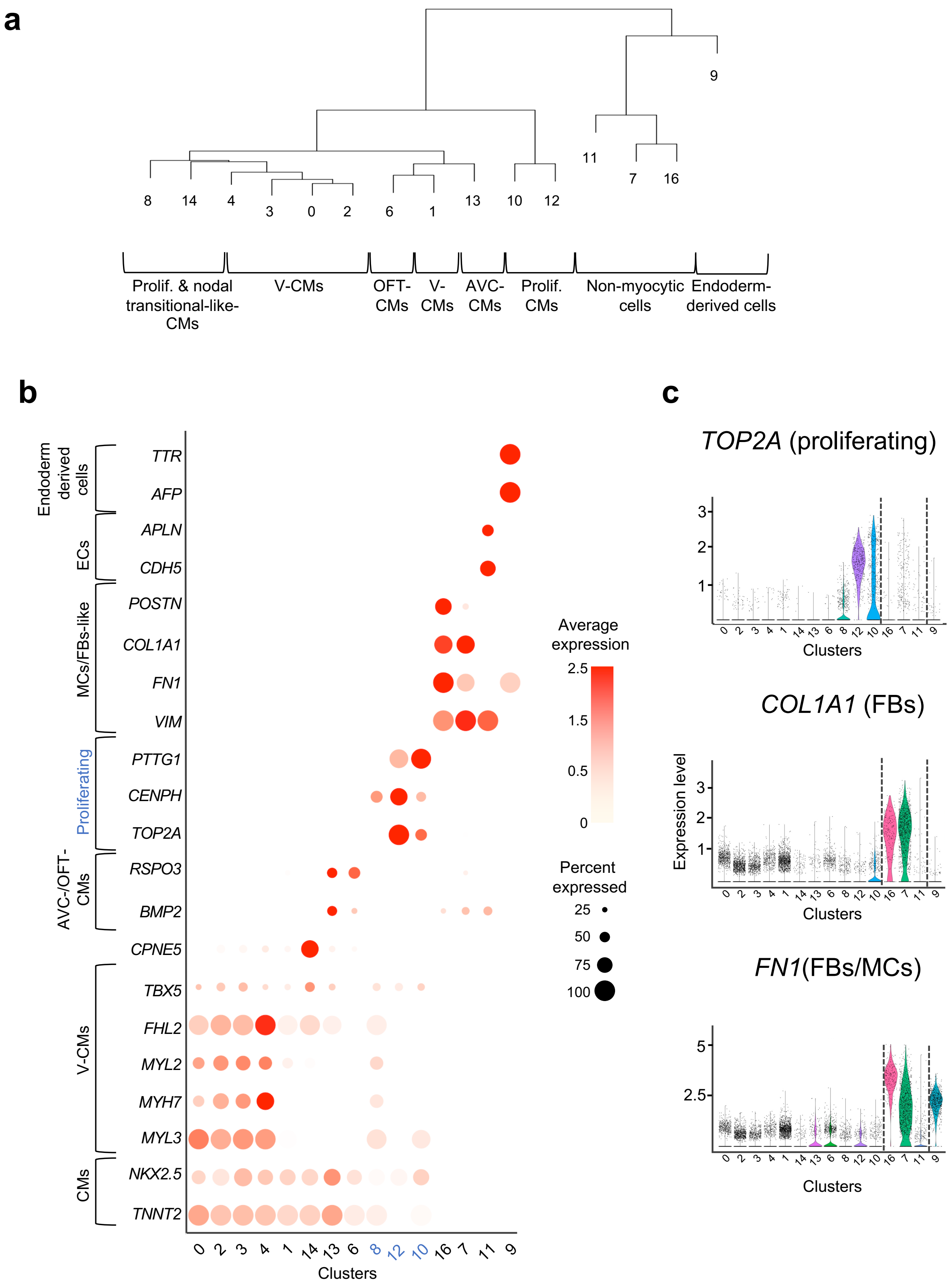

Supplementary Figure 8: Characterization of CPC-RA- and CPC-RA+ derived cells (related to Figure 5). (a) Dendrogram showing hierarchical clustering of the averaged, corrected, normalized expression values per indicated day 30 scRNA-seq clusters. UMAP plot shown in Fig. 5b. **(b)** Dot plot showing the expression level of selected differentially expressed genes for indicated clusters at day 30 in the UMAP plot shown in Fig. 5b. Proliferating clusters

are in blue. **(c)** Violin plots showing expression levels of the proliferation marker *TOP2A*, the fibroblast markers *FN1* and *COL1A1* in the scRNA-seq clusters shown in Fig. 5b*.* FBs: fibroblasts; MCs: mesenchymal cells.

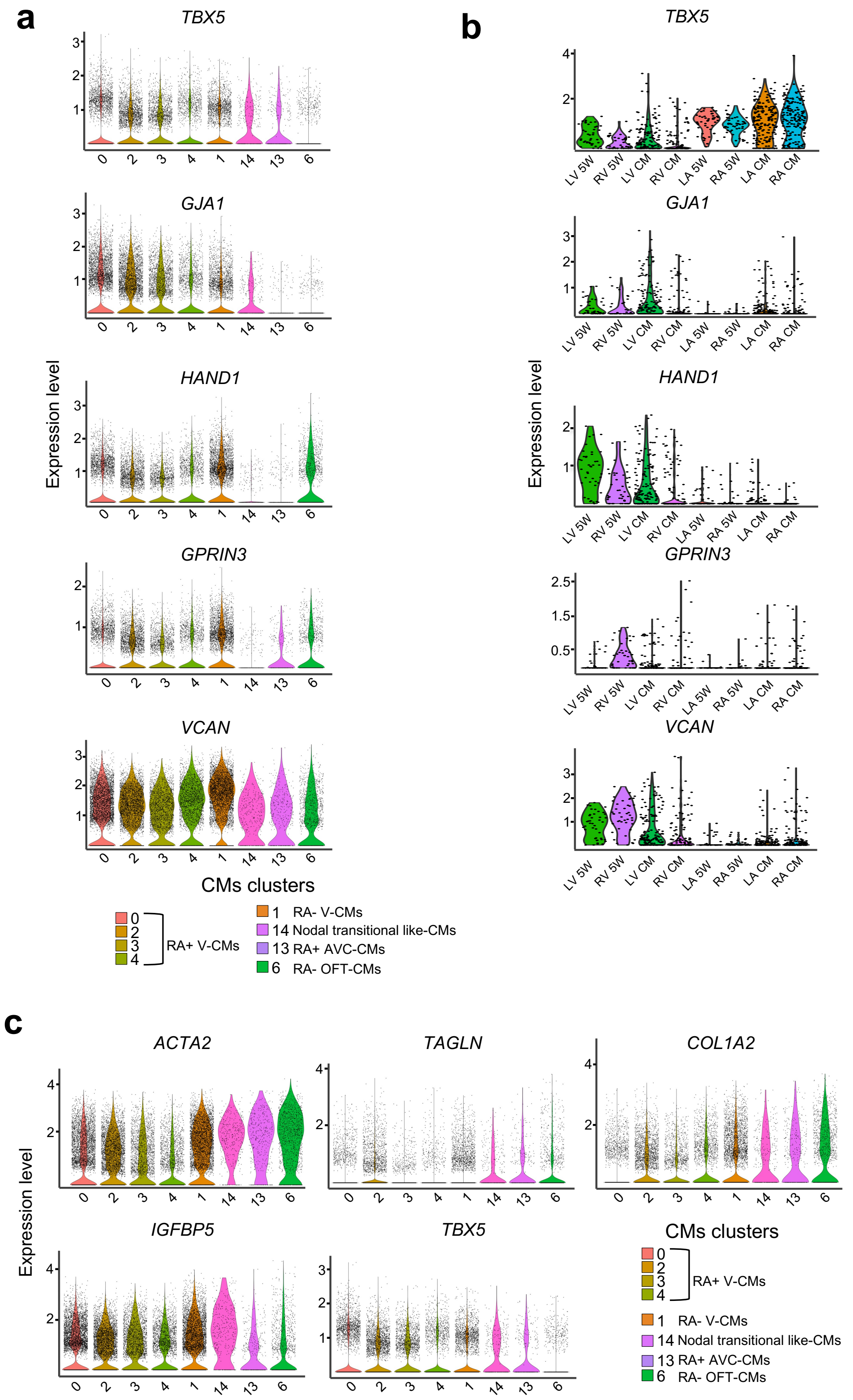

Supplementary Figure 9: CPC-RA- and CPC-RA+ contribute to different cardiomyocyte subtypes clusters (related to Figure 5). (a) Violin plots showing the expression levels of markers enriched in LV- and RV-like CMs at day 30 in the UMAP plot shown in Fig. 5b. **(b)**

Violin plots showing expression of markers enriched in indicated heart compartments at different stages of fetal human heart development from Cui et al.[25]. LV: left ventricle; RV: right ventricle; LA: left atria; RA: right atria; 5w: 5th week of gestation. CM: cardiomyocytes; for this plot only data from early fetal hearts (5-7th weeks of gestation) were considered. **(c)** Violin plots showing the expression levels of outflow tract cardiomyocytes markers (*ACTA2, COL1A2, TAGLN, BMP2*) and *TBX5* at day 30 in the UMAP plot shown in Fig. 5b.

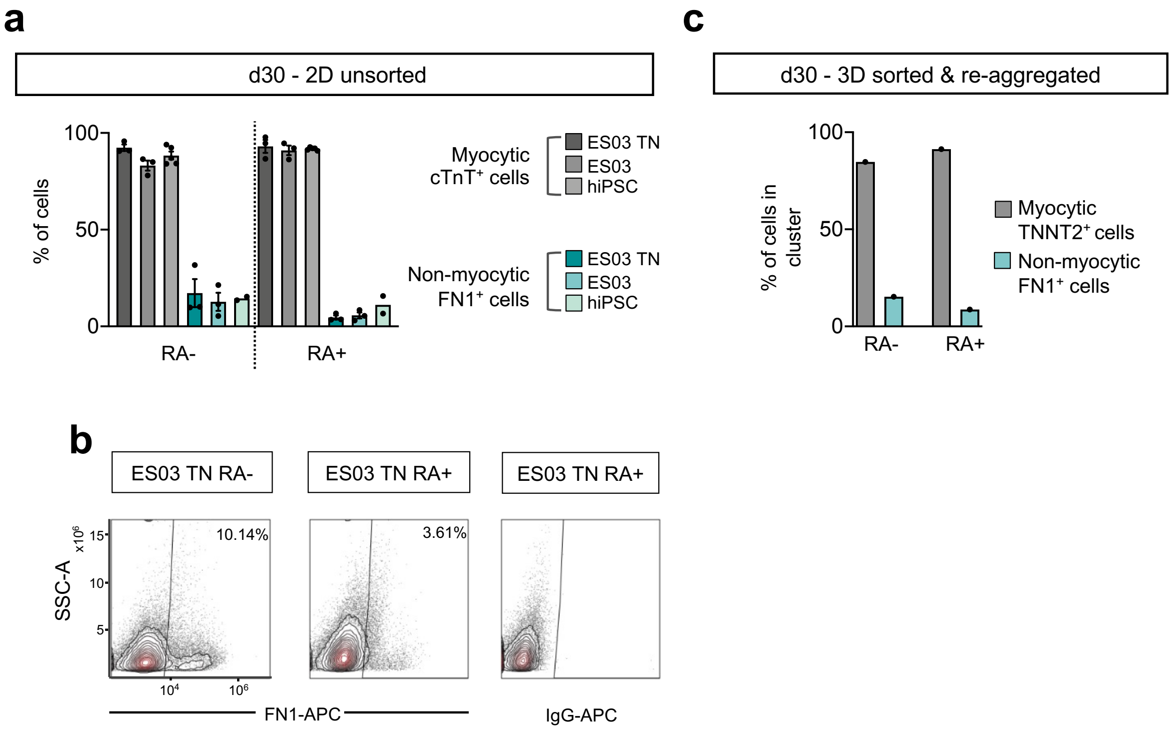

Supplementary Figure 10: Differentiation efficiency into the myocytic lineages of sorted and unsorted CPCs is similar. (a) Quantification of flow cytometry analysis at day 30 of cells stained positive for FN1 or cTnT within RA- differentiation or 0.5 µM 4d RA differentiation for 3 hPSC lines. Data are mean ± SEM; n = 3 independent experiments; except for hiPSC cTnT n = 5, and FN1 data are mean n = 2. **(b)** Representative plots of flow cytometry analysis of cells stained positive for FN1 or IgG (negative control) at day 30 of differentiation without retinoic acid (RA-) and with 0.5 µM RA for 4d (RA+) of ES03 TN cell line. Related to Supplementary Figure 10a. **(c)** Quantification of cells in myocytic clusters ($TNNT2^{+}$) or non-myocytic clusters ($FN1^{+}$) within day 30 cells derived from CPC populations obtained during differentiation without (RA-) and with 0.5 µM RA for 4d (RA+). Source data are provided as a Source Data file.

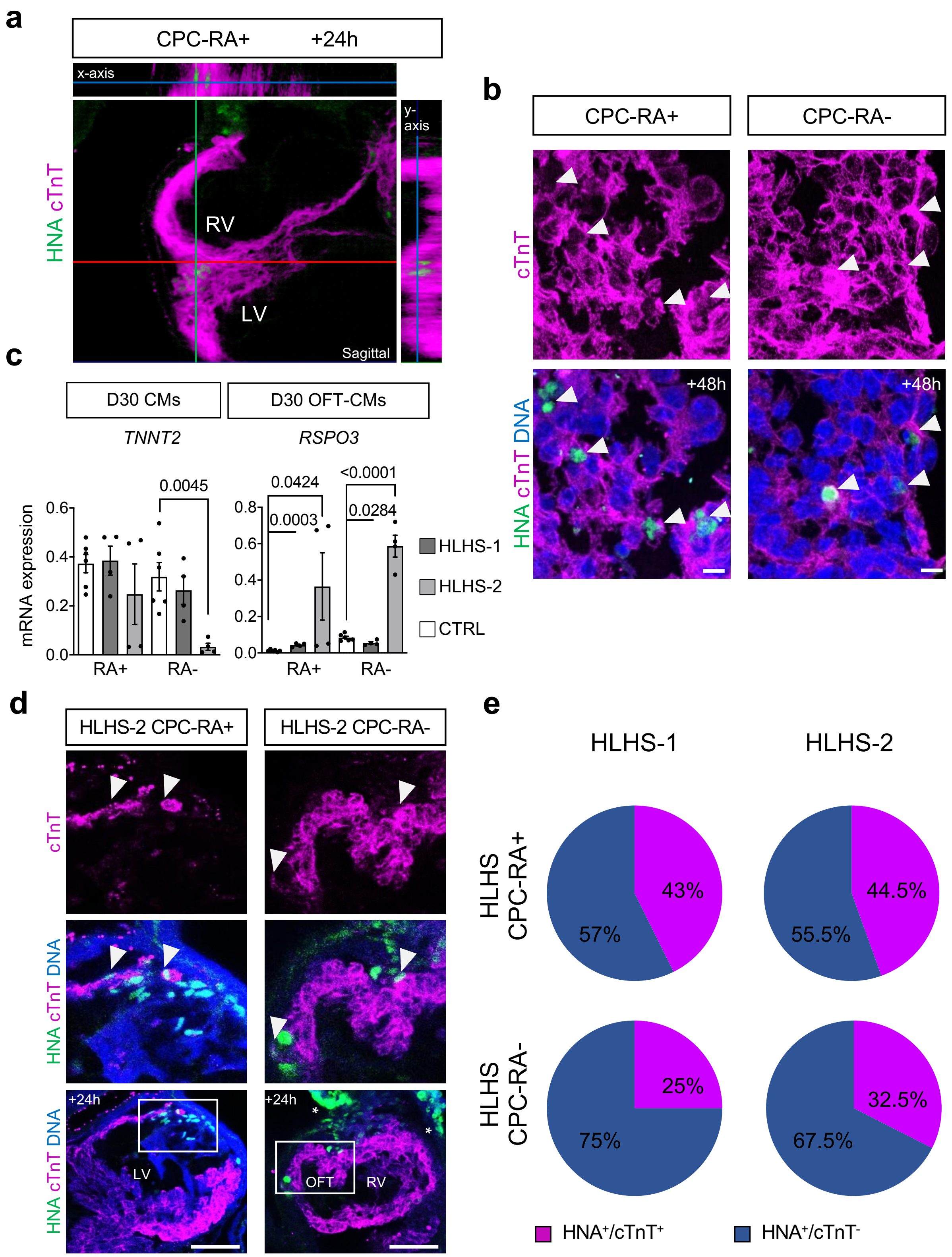

Supplementary Figure 11: Validation of CPC-RA- and CPC-RA+ differentiation potential *ex-vivo* and utilization of heart field specific protocol in disease modelling (related to Figure 6 and 7). (a) Sagittal view of the heart region (marked by cTnT in magenta) of a representative whole mount embryo injected with CPC-RA+ (HNA) after 24h of culture *ex vivo*. The red horizontal line shows location of the section view of the x-axis on the top. The green

vertical line depicts location of the section view of the y-axis on the right side. Blue lines indicate location of the z-axis. LV: left ventricle; RV: right ventricle. **(b)** CPC-RA+ and CPC-RA- stained positive for sarcomeric cTnT following injection into the murine embryo heart. **(top)** single channel image for cTnT (magenta) of the murine myocardium with integrated CPC-RA+ or CPC-RA-. **(bottom)** corresponding merged images shown in figure 6f. Arrowheads indicate human cells marked by HNA (green). Scale bars = 10µm. Nuclei were counterstained with Hoechst-33258 (blue). **(c)** mRNA expression of *TNNT2* (relative to *GAPDH*) and *RSPO3* (relative to *GAPDH* and *TNNT2*) at day 30 of RA- or RA+ (0.5 µM 4d) differentiation of HLHS patients-derived hiPSCs (HLHS-1, HLHS-2) and healthy control hiPSCs (CTRL). Data are mean ± SEM; for HLHS-1 and HLHS-2: n = 3 differentiations. CTRL values depict data from two independent cell lines, for each n = 3 differentiations. Exact p-values of unpaired two-tailed *t*-test are shown**. (d)** Representative immunofluorescence images of the heart region (marked by cTnT in magenta) of embryos injected with HLHS-derived CPC-RA+ and HLHS-derived CPC-RA- (marked by HNA) after 24h of culture *ex vivo*. Arrowheads indicate human cells marked by HNA (green). Nuclei were counterstained with Hoechst-33258 (blue). **(top, middle)** A high magnification of boxed regions from the corresponding bottom panels. Stars indicate unspecific stain of the yolk sac. Scale bars = 50 µm. **(e)** Quantification of the percentage of human CMs ($HNA^+/cTnT^+$) and non-CMs ($HNA^+/cTnT^-$) found within the mouse heart following injection of HLHS-derived CPC-RA+ or HLHS-derived CPC-RA- from HLHS-1 and -2. Percentage represents mean of each condition. For HLHS-derived CPC-RA+ from HLHS-1 n = 3 independent embryos and from HLHS-2 n = 2 independent embryos. For HLHS-derived CPC-RA- from HLHS-1 n = 2 independent embryos and from HLHS-2 n = 2 independent embryos. Source data are provided as a Source Data file.
