# Supplementary material for: Retinoic acid signaling modulation guides in vitro specification of human heart field-specific progenitor pools
Source: Nat Commun. 2023 Apr 3;14:1722. doi: 10.1038/s41467-023-36764-x (PMC10070453; doi:10.1038/s41467-023-36764-x)
Supplement: Supplementary file 3 — Description of Additional Supplementary Files [file 41467_2023_36764_MOESM3_ESM.pdf]

Description of Additional Supplementary Files

File Name: Supplementary Data 1
Description: Parameters of action potential traces. Related to figure 2.
Sheets 1-4 show parameters characterizing action potential traces obtained through whole-cell voltage clamp recording at d30 of differentiation without RA (sheets 1, 3) or with 1 µM RA for 4d (sheets 2, 4) for each measured cells: Maximum Diastolic Potential (MDP), Amplitude, and Delta; all corrected for Liquid Junction Potential (LJPc) as well as action potential duration (APD); APD at 50% (APD50) and 90% (APD90) and their ratio; frequency of beating (registered beats per minute) obtained through whole-cell voltage clamp recording. Classification of cardiomyocyte subtypes was done based on APD90/50 ratio.

File Name: Supplementary Data 2
Description: List of differentially expressed genes from the combined analysis of d1.5 and d4.5. Related to Figure 3.
Sheet 1 shows differentially expressed genes of single cells captured at d1.5, and sorted cells at d4.5; merged samples of differentiations with 0.5 µM RA for 4d (RA+) and without RA (RA-). UMAP plot shown in Fig. 3.

File Name: Supplementary Data 3
Description: List of differentially expressed genes used to identify surface markers for the JCF. Related to Figure 4.
Sheet 1 shows differentially expressed genes (75% significance cut-off) of cluster Me5 coming from publicly available datasets of Tyser et al. (2021) that are classified as genes coding for cell surface proteins using Toppgene website (toppgene.cchmc.org). Related to Figure 4.
Sheet 2 shows overlapping differentially expressed genes classified as genes coding for cell surface protein between Me5 (75% significane; Tyser et al., 2021) and differentially expressed genes of clusters 14 and 18 of single cells captured at d1.5, and sorted cells at d4.5; merged samples of differentiations with 0.5 µM RA for 4d (RA+) and without RA (RA-). Related to Figure 4.

File Name: Supplementary Data 4
Description: Summary of analysis of differentiation potential of $eGFP^{+}/ITGA8^{+}$ and $eGFP^{+}/ITGA8^{-}$ cells. Related to Figure 4.
Summary of quantification of d14 $eGFP^{+}/ZO1^{+}$ EpiCs and $eGFP^{+}/cTnT^{+}$ CMs clusters that differentiated from sorted at d4.5 $eGFP^{+}/ITGA8^{+}$ and $eGFP^{+}/ITGA8^{-}$ cells

File Name: Supplementary Data 5
Description: List of differentially expressed genes from d30. Related to Figure 5.
Sheet 1 shows differentially expressed genes of single cells captured at d30; merged samples of differentiations with 0.5 µM RA for 4d (RA+) and without RA (RA-). Related to Figure 5. UMAP plot shown in 5b.

File Name: Supplementary Data 6
Description: List of differentially expressed genes between indicated ventricular CMs clusters from d30. Related to Figure 5.

Differentially expressed genes between indicated clusters at d30. Related to Figure 5b. Genes upregulated and downregulated in cluster 1 relative to indicated clusters.

File Name: Supplementary Data 7
Description: List of differentially expressed genes from the subclustering of the non-myocytic cells from the analysis of d30. Related to Figure 5.
Sheet 1 shows differentially expressed genes of the second round of clustering of non-myocytic clusters of single cells captured at d30 cells obtained by reaggregation of sorted cells on d4.5; merged samples of differentiations with 0.5 µM RA for 4d (RA+) and without RA (RA-). Related to Figure 4. UMAP lot shown in 5g.

File Name: Supplementary Data 8
Description: Summary of mouse embryo experiments.
Sheet 1 shows number and percentage of total embryos injected with ES03 TN CPC-RA+ and ES03 TN CPC-RA- as well as not successfully injected or malformed embryos after culture ex vivo, which were excluded for quantification in figure 6f.
Sheet 2 shows number and percentage of cTnT+/HNA+ or cTnT-/HNA+ in ES03 TN or HLHS-derived CPC-RA+ and RA- used for quantification in Fig. 6i and Fig. 7h, respectively. For calculation of significance among groups the unpaired t test was performed.
Sheet 3 shows number and percentage of cTnT+/HNA+ or cTnT-/HNA+ in HLHS-1 and HLHS-2 derived CPC-RA+ and RA- used for quantification in Supplementary Figure 11.

File Name: Supplementary Data 9
Description: Primary antibodies used for immunofluorescence/FACS staining.

File Name: Supplementary Data 10
Description: Secondary antibodies used for immunofluorescence/FACS staining.

File Name: Supplementary Data 11
Description: Sequences of primers used for qPCR.

File Name: Supplementary Movie 1
Description: Representative 3D reconstruction of an *ex-vivo* cultured mouse embryo after immunofluorescent staining with cTnT (magenta) and Hoechst 33258 (blue). Cardiac regions that were used to assess location of injected human CPCs in the heart are annotated.
