# Supplementary material for: Retinoic acid signaling modulation guides in vitro specification of human heart field-specific progenitor pools
Source: Nat Commun. 2023 Apr 3;14:1722. doi: 10.1038/s41467-023-36764-x (PMC10070453; doi:10.1038/s41467-023-36764-x)
Supplement: Supplementary file 16 — Reporting Summary [file 41467_2023_36764_MOESM16_ESM.pdf]

nature portfolio

Corresponding author(s): Alexander Goedel, Alessandra Moretti, Karl-Ludwig Laugwitz

Last updated by author(s): Feb 6, 2023

Reporting Summary

Nature Portfolio wishes to improve the reproducibility of the work that we publish. This form provides structure for consistency and transparency in reporting. For further information on Nature Portfolio policies, see our Editorial Policies and the Editorial Policy Checklist.

Statistics

For all statistical analyses, confirm that the following items are present in the figure legend, table legend, main text, or Methods section.

n/a	Confirmed	
☐	☒	The exact sample size (*n*) for each experimental group/condition, given as a discrete number and unit of measurement
☐	☒	A statement on whether measurements were taken from distinct samples or whether the same sample was measured repeatedly
☐	☒	The statistical test(s) used AND whether they are one- or two-sided<br>*Only common tests should be described solely by name; describe more complex techniques in the Methods section.*
☒	☐	A description of all covariates tested
☐	☒	A description of any assumptions or corrections, such as tests of normality and adjustment for multiple comparisons
☐	☒	A full description of the statistical parameters including central tendency (e.g. means) or other basic estimates (e.g. regression coefficient) AND variation (e.g. standard deviation) or associated estimates of uncertainty (e.g. confidence intervals)
☐	☒	For null hypothesis testing, the test statistic (e.g. *F*, *t*, *r*) with confidence intervals, effect sizes, degrees of freedom and *P* value noted<br>*Give P values as exact values whenever suitable.*
☒	☐	For Bayesian analysis, information on the choice of priors and Markov chain Monte Carlo settings
☒	☐	For hierarchical and complex designs, identification of the appropriate level for tests and full reporting of outcomes
☒	☐	Estimates of effect sizes (e.g. Cohen's *d*, Pearson's *r*), indicating how they were calculated

Our web collection on statistics for biologists contains articles on many of the points above.

Software and code

Policy information about availability of computer code

Data collection	Leica application suite from Leica Microsystems, confocal laser scanning microscopy (Leica Microsystems, SP8), THUNDER system (Leica Mircrosystems)<br>Axopatch 200B amplifier (Molecular Devices, USA)<br>FACSAria III (BD)<br>CytoFlex S (Beckman Coulter)<br>Gallios (Beckman Coulter)
Data analysis	Leica Application Suite X software v3.5.7.23225<br>GraphPad Prism V5 and V8<br>ImageJ V1.53a<br>Kaluza V2.1 (Beckman Coulter)<br>Clampfit software (v10.4 and v10.7) (Molecular Devices, USA).<br>For scRNA-Seq analysis we used: the R statistical programming language (v3.5.1), Cell Ranger pipeline (v6.1.1), Seurat (v4.0.1 and v4.1.1) (Hao, Y. et al. Integrated analysis of multimodal single-cell data. Cell 184, 3573-3587.e29 (2021)), clustree package (v0.4.3), yaGST R package (v2017.08.25) (https://rdrr.io/github/miccec/yaGST/) (Pagnotta, S. M. Competitive gene set and regulon tests. R package version 2017.08.25. (2017)), to impute missing values we used ALRA as implemented in the Seurat package v4.1.1 (Linderman GC et al., Zero-preserving imputation of single-cell RNA-seq data. Nat Commun. 11;13(1):192., doi: 10.1038/s41467-021-27729-z. (2022)).

For manuscripts utilizing custom algorithms or software that are central to the research but not yet described in published literature, software must be made available to editors and reviewers. We strongly encourage code deposition in a community repository (e.g. GitHub). See the Nature Portfolio guidelines for submitting code & software for further information.

Data

Policy information about availability of data

All manuscripts must include a data availability statement. This statement should provide the following information, where applicable:

- Accession codes, unique identifiers, or web links for publicly available datasets
- A description of any restrictions on data availability
- For clinical datasets or third party data, please ensure that the statement adheres to our policy

The scRNA-seq data generated in this study have been deposited in the GEO database under accession code GSE197660 [https://www.ncbi.nlm.nih.gov/geo/query/acc.cgi?acc=GSE197660]. The raw scRNA-seq data by Zhang et al.5 is available in the GEO database under accession code GSE176306 [https://www.ncbi.nlm.nih.gov/geo/query/acc.cgi?acc=GSE176306]. The processed data was downloaded from the University of California, Santa Cruz (UCSC) cell browser https://cells.ucsc.edu/?ds=chi-10x-mouse-cardiomyocytes. The raw scRNA-seq data by Tyser et al.6 is available in the European Nucleotide Archive database under study PRJEB14363 [https://www.ebi.ac.uk/ena/browser/view/PRJEB14363], and ArrayExpress, under accession E-MTAB-7403 [https://www.ebi.ac.uk/biostudies/arrayexpress/studies/E-MTAB-7403]. The processed data was downloaded from the Cancer Research UK Cambridge Institute https://content.cruk.cam.ac.uk/jmlab/mouseEmbryonicHeartAtlas/. The raw and processed scRNA-seq data by Ivanovitch et al.4 is available in the GEO database under accession code GSE153789. All the data generated in this study are either deposited in the above-mentioned repository or are provided in the Supplementary Information and Source Data file.

Human research participants

Policy information about studies involving human research participants and Sex and Gender in Research.

Reporting on sex and gender	There were no human research participants in this study.
Population characteristics	Not applicable.
Recruitment	Not applicable.
Ethics oversight	Not applicable.

Note that full information on the approval of the study protocol must also be provided in the manuscript.

Field-specific reporting

Please select the one below that is the best fit for your research. If you are not sure, read the appropriate sections before making your selection.

☒ Life sciences ☐ Behavioural & social sciences ☐ Ecological, evolutionary & environmental sciences

For a reference copy of the document with all sections, see nature.com/documents/nr-reporting-summary-flat.pdf

Life sciences study design

All studies must disclose on these points even when the disclosure is negative.

Sample size	Sample size was not pre-determined. All experiments were designed to generate 3 biological independent samples for every conditions since this is generally assumed to allow assessing relevant differences between samples (with exception of scRNA-Seq experiment). In cases when less, or more samples were generated, all available samples were reported and the exact number of replicates is stated alongside the data presented. Statistical analysis was only performed in cases, where at least 3 biological independent samples were available for each group.
Data exclusions	Mouse-human embryo chimeras experiments: Embryos with visual damage after removal from the uterine cavity were excluded directly. Undamaged embryos were injected with hCPCs and cultured ex vivo as described in the methods. Embryos failing to develop properly ex-vivo were excluded from the analysis.<br>3D culture of sorted cells: experiments were excluded in case of failure of sorting; failure was defined as relevant contamination with cell populations deviating from the expected fluorescence pattern as assessed by microscope imaging of fluorescent markers 24 h after sorting or high abundance of dead cells
Replication	The reproducibility was validated in 4 independent hPSC lines (2 hESC-lines and 2 hiPSC-lines). All experiments, except scRNA-seq analysis, was repeated at least three times, if not stated otherwise. The exact number of repetitions are stated in the respective figure legends.
Randomization	Monolayer differentiations and 3D culture were conducted in 24-well and 96-well plate format, respectively. Wells were randomly assigned for treatment/downstream analyses: at day 2 wells were randomly assigned for treatment with retinoic acid; wells that did not meet exclusion criteria were randomly assigned for collection of material for RNA/qPCRs; wells were randomly assigned for dissociation followed by flow cytometry analysis or dissociation followed by immunofluorescence analysis. Wells for immunofluorescence analysis were randomly assigned to be stained with antibodies. For mouse-human embryo chimeras experiments, mouse embryos were randomly assigned to experimental groups (injection with hCPCs-RA- or hCPCs-RA+ - wells with hCPCs for injection were also randomly assigned).
Blinding	For the analysis of the qPCR data, blinding was not necessary since this analysis is observer independent and all generated values from all

Blinding

experiments that did not meet exclusion criteria were reported. For the analysis of immunofluorescent images of the in vitro culture blinding was not feasible since cells from different conditions were identifiable based on different appearance. For the quantification of the immunofluorescent images of the eGFP+/ITGA8+ or eGFP+ITGA8- cells stained for epicardial or myocardial markers, the person performing the quantification was blinded to the condition by pseudonymization of the samples. The person analyzing the immunofluorescent images for quantification and identification of the location of human cells injected into mouse embryos was also blinded to the condition by pseudonomization of the samples.

Reporting for specific materials, systems and methods

We require information from authors about some types of materials, experimental systems and methods used in many studies. Here, indicate whether each material, system or method listed is relevant to your study. If you are not sure if a list item applies to your research, read the appropriate section before selecting a response.

Materials & experimental systems

n/a	Involved in the study
☐	☒ Antibodies
☐	☒ Eukaryotic cell lines
☒	☐ Palaeontology and archaeology
☐	☒ Animals and other organisms
☒	☐ Clinical data
☒	☐ Dual use research of concern

Methods

n/a	Involved in the study
☒	☐ ChIP-seq
☐	☒ Flow cytometry
☒	☐ MRI-based neuroimaging

Antibodies

Antibodies used

anti-Rabbit Donkey Alexa Fluor 647 Invitrogen A31573 1:500
anti-Mouse Donkey Alexa Fluor 647 Invitrogen A31571 1:500
anti-Chicken Donkey Alexa Fluor 488 Jackson Immuno Research 703-545-155 1:500
anti-Goat Donkey Alexa Fluor 594 Invitrogen A11058 1:500
anti-Mouse Goat Alexa Fluor 488 Invitrogen A11001 1:500
anti-Rabbit Goat Alexa Fluor 594 Invitrogen A11012 1:500
anti-Mouse Donkey Alexa Fluor 594 Invitrogen A21203 1:500
anti-Mouse Goat Pacific Blue Invitrogen P31582 1:500
anti-CK-18 Mouse Abcam ab668 1:100
anti-cTnT Rabbit Abcam ab92546 1:500
anti-Fibronectin Rabbit Abcam ab2413 1:250
anti-ISL1 Mouse Developmental Studies, Hybridoma Bank 39.4D5 1:100
anti-NKX2.5 Rabbit Novus Biologicals NBP1-31558 1:200
anti-TCF21 Rabbit Sigma-Aldrich HPA013189 1:100
anti-eGFP Chicken Abcam ab13970 1:500
anti-rabbit IgG isotype control Rabbit Abcam ab27478 (for flow cytometry analysis: 1 µg of antibody per 100 µl of buffer per 1 000 000 cells)
anti-mCherry Goat Sicgen ab0040-200 1:200
anti-TBX5 Rabbit Abcam ab18531-100 1:100
anti-mouse IgG2b isotype control Mouse Sigma Aldrich M5534 (for flow cytometry analysis: 1 µg of antibody per 100 µl of buffer per 1 000 000 cells)
anti-ITGA8-Alexa-Fluor-647-conjugated Mouse R&D systems fab9194r for flow cytometry analysis: 10 µl of antibody per 100 µl of buffer per 1 000 000 cells); in the manuscript identified as: ITGA8-APC
anti-ZO-1 Mouse Invitrogen 33-9100 1:100
anti-Human nuclear antigen (HNA) Mouse Sigma Aldrich MAB1281 1:200
anti-COUP-TF II/NR2F2 Mouse R&D systems PP-H7147-00 1:300
anti-Myosin Light Chain 2/MLC-2V Rabbit Proteitech 10906-1-AP 1:100
anti-Myosin light chain 2/MLC-2A Mouse Synaptic Systems 311 011 1:200
anti-WT1 Rabbit Abcam ab89901 1:100
anti-mouse IgG-APC-conjugated Miltenyi Biotec 130-120-495 1:50

Validation

All primary antibodies were validated for immunocytochemistry/immunohistochemistry analysis of human samples by the respective manufacturers. All secondary antibodies were validated for immunocytochemistry/immunohistochemistry by the respective manufacturers.

Eukaryotic cell lines

Policy information about cell lines and Sex and Gender in Research

Cell line source(s)

hiPSC CTRL line was previously generated in house and has been deposited in the cell register hPSCreg MRIi003-A
hIPSC eGFP+ line was previously generated in house and has been deposited in the cell register hPSCreg MRIi003-A-8
hiPSC HLHS-1 line was previously generated in house as described in Krane et al. 2021, Circulation (10.1161/CIRCULATIONAHA.121.056198) and has been deposited in the cell register hPSCreg MRIi018-A
HLHS-2 line was generated in German Heart Centre (Munich, Germany) as described in Krane et al. 2021, (Circulation

(10.1161/CIRCULATIONAHA.121.056198), and has been deposited in the cell register hPSCreg DHMi003-A
The ES03, ES0-NKX2.5-eGFP line was generously provided by Dr. David Elliott (MCRI, Australia). ES03 line (hPSCreg ESIBIe003) was generated by ES Cell International Pte Ltd in Singapore.
The ES03-TN line was generated in the course of this research project. The generation of the line is described in detail in the manuscript.

Authentication

The pluripotency, trilineage potential, and karyotype of hiPSC lines were validated in previous studies (Moretti et al., 2020, Nat Med, 10.1038/s41591-019-0738-2; Krane et al. 2021, Circulation, 10.1161/CIRCULATIONAHA.121.056198). The ES03, ES03-NKX2.5-eGFP line was validated by Dr. David Elliott (Elliott et al., 2011, Nat Meth, 10.1038/nmeth.1740). The hES03-TN was validated in this research project. The parental and modified ES03 lines were distinguished from hiPSCs lines based on characteristic morphology of the undifferentiated cells in culture. The genotypes (GFP, mCherry) of the different hiPSC and ES03 lines were distinguished based on characteristic fluorescent expression during differentiation or constitutive eGFP expresssion, respectively.

Mycoplasma contamination

All cell lines used tested negative for mycoplasma.

Commonly misidentified lines
(See ICLAC register)

No commonly misidentified lines were used.

Animals and other research organisms

Policy information about studies involving animals; ARRIVE guidelines recommended for reporting animal research, and Sex and Gender in Research

Laboratory animals

For this study, we used a mouse line with C57/B6 background. C57BL/6 male (2-6 months) and female (2-3 months) mice were housed at 20–24°C, 45–60% humidity, and a dark/light cycle of 12/12h.

Wild animals

No wild animals were used in this study.

Reporting on sex

Injected embryos were not selected for sex.

Field-collected samples

No field-collected samples were used in this study.

Ethics oversight

All animal experiments were performed in accordance with German animal protection laws and EU ethical guidelines (Directive 2010/63/EU). Pregnant females sacrificed for embryo collection within the first two-thirds of gestation are reported to the inspection authority as organ collection and do not need additional ethical approvement.

Note that full information on the approval of the study protocol must also be provided in the manuscript.

Flow Cytometry

Plots

Confirm that:

☒ The axis labels state the marker and fluorochrome used (e.g. CD4-FITC).

☒ The axis scales are clearly visible. Include numbers along axes only for bottom left plot of group (a 'group' is an analysis of identical markers).

☒ All plots are contour plots with outliers or pseudocolor plots.

☒ A numerical value for number of cells or percentage (with statistics) is provided.

Methodology

Sample preparation

For flow cytometry based sorting and analysis, ES03 TN derived differentiated cells between d0-d8 of differentiation were washed two times with PBS(-/-), dissociated with Accutase (A11105013, Gibco/Invitrogen 5min, 37°C), centrifuged at 1200 rpm for 5min, resuspended in 2% FCS in PBS, filtered through a 40 µm filter. From day 8 on cells were subjected to papain-based dissociation (Fischer et al, 2018, Stem Cell Res). DAPI (D3571, ThermoFisher Scientific) staining was used to discriminate dead and live cells (final concentration 0.01 ng/µl). For intracellular/intranuclear staining for flow cytometry cells were dissociated, counted and distributed in equal numbers per sample, fixed with 4% PFA, and subjected to staining preocedure. For flow cytometry analysis of surface protein-stained cells, cells were dissociated, counted and distributed in equal numbers per sample, and subjected to staining procedure.

Instrument

FACSAria III (BD)
CytoFlex S (Beckman Coulter)
Gallios (Beckman Coulter)

Software

Kaluza V2.1 (Beckman Coulter)

Cell population abundance

Purity of sorted and further aggregated populations expressing mCherry+, eGFP+, double eGFP+/mCherry+ was assesed next day by microscope imaging of fluorescent markers.

Gating strategy

For sorting experiments: gate including cells and excluding debris was assigned, followed by gate to include live cells only.

Gating strategy

Gate for cells negative for endogenously expressed mCherry and eGFP was assigned at d0; for cells stained for ITGA8-647 surface marker, several negative controls were used including: d5 CPCs unstained control; d5 CPCs IgG-647 control; d0 ITGA8-647 to assign the border between negative/positive populations.
For live time course analysis: gate for cells negative for endogenously expressed mCherry and eGFP was assigned at d0.
For intracellular/intranuclear staining no-primary antibody, no-secondary antibody, IgG antibody controls were performed to assign the border between negative/positive populations. Additionally, stained d0 cells were used to adjust gating.

☒ Tick this box to confirm that a figure exemplifying the gating strategy is provided in the Supplementary Information.
